# Genetic profile of Chinese patients with small bowel cancer categorized by anatomic location

**DOI:** 10.1186/s12920-023-01736-z

**Published:** 2023-11-16

**Authors:** Chengmin Shi, Junrui Ma, Tong Zhang, Yanqiang Shi, Weiming Duan, Depei Huang, Hushan Zhang, Yujian Zeng

**Affiliations:** 1 Department of Gastrointestinal Surgery, The First Affiliated Hospital of Kunming Medical University, No 295, Xichang Road, Kunming, Yunnan Province 650032 P.R. China; 2 School of Nursing, Yunnan University of Traditional Chinese Medicines, Kunming, Yunnan 650504 P.R. China; 3 The Medical Department, 3D Medicines Inc., Building 2, Block B, 158 XinJunhuan Street, Pujiang Hi-Tech Park, MinHang District, Shanghai, 201114 P.R. China; 4 Zhaotong Health Vocational College, Zhaotong, Yunnan 657000 P.R. China

**Keywords:** Small bowel cancer, Next-generation sequencing, Genes

## Abstract

**Background:**

Small bowel cancer (SBC) is a very rare solid malignancy. Consequently, compared with other malignant gastrointestinal tumors, our knowledge regarding SBC, specifically its molecular attributes, remains limited. Herein, we aim to provide an overview of the gene characteristics of Chinese patients with SBC, We particularly focus on elucidating the genetic intricacies that differentiate SBC patients whose primary tumors originate in distinct anatomical regions within the small bowel.

**Methods:**

During the period ranging from February 2018 to December 2022, a total of 298 tumor samples were consecutively collected from Chinese patients diagnosed with small bowel cancer.. Next-generation sequencing (NGS) was performed to detect gene mutation, assess microsatellite instability (MSI), and evaluate tumor mutational burden (TMB). Additionally,, IHC was used to analyze the level of PD-L1 expression within the samples.

**Results:**

The outcomes of the next-generation sequencing (NGS) unveiled the predominant gene mutations observed in Chinese patients with small bowel cancer (SBC). The top ten gene mutations identified were as follows: *TP53* (53%), *KRAS* (51%), *APC* (31%), *SMAD4* (19%), *VEGFA* (15%), *CDKN2A* (15%), *RAC1* (15%), *LRP1B* (14%), *MGMT* (14%, *CD74* (13%). Subsequent analysis revealed disparities in the gene landscape between the cohort in this study and that of the Memorial Sloan Kettering Cancer Center (MSKCC), Notably, distinguishable mutational frequencies were identified in several genes, including *ERBB2, FBXW7, PIK3CA, *etc*. which* exhibited contrasting presence in both this cohort and the MSKCC cohort.. Furthermore, we noticed variations in the frequency of gene mutations among SBC patients depending on the specific anatomical site where the tumors originated within the small bowel. In addition, the distribution of patients with high microsatellite instability (MSI-H) and tumor mutational burden (TMB) levels varied among SBC patients with tumors originating from the duodenum, jejunum, and ileum.

**Conclusion:**

Chinese patients with small bowel cancer exhibited a distinct genetic profile in comparison to other populations, highlighting a unique genetic landscape. Furthermore, noticeable disparities in the genetic landscape were observed between patients with cancer situated in the duodenum and those with cancer affecting other regions of the small bowel, this suggests that these patients should be treated differently.

**Supplementary Information:**

The online version contains supplementary material available at 10.1186/s12920-023-01736-z.

## Introduction

Small bowel cancer (SBC) is very rare and has been reported to account for approximately 5% of all gastrointestinal malignancies [1], but its incidence has been increasing in the last decade [2].

The main histological types of SBC are adenocarcinomas, sarcomas, neuroendocrine tumors, gastrointestinal stromal tumors, etc. Adenocarcinoma is the most common SBC histologic type [3]. Due to its heterogeneity and rarity, a majority of cases are typically diagnosed and confirmed at relatively advanced stages, leading to suboptimal treatment options and prognosis.. Besides, the existing medical evidence for SBC is predominantly derived from studies with limited sample sizes or retrospective analyses. In addition to chemotherapy, the NCCN guidelines recommend the utilization of immune checkpoint inhibitors (ICIs) and antiangiogenic agents as viable treatment options for both duodenum and jejunum/ileum cancers. In recent years, there has been a significant surge in the significance of next-generation sequencing (NGS) technology for analysis of molecular characteristics and prognostic value of specific mutated genes in diverse solid tumors. Multiple factors have been observed to be connected with the effectiveness of ICIs in various solid cancers, including colorectal cancer, gastric cancer, lung cancer, and some other solid cancers. These factors encompass the microsatellites (dMMR/MSS), level of tumor mutational burden (TMB), level of PD-L1 expression, and the existence of specific gene mutations [4, 5]. However, research on the genetic landscape in SBC was very limited for Chinese patients, and the results of molecular characteristics displayed were varied in previous reports with different sample sizes [6, 7]. As of recently, we have only found one report on the molecular characteristics of Chinese patients with SBC in PubMed, and it collected data from only 76 patients [8]. Herein, we present our findings on the molecular characteristics exhibted by Chinese individuals diagnosed with SBC. Our insights are built upon an extensive dataset of 298 SBC patients. Furthermore, we conducted a comparative analysis to discern the disparities in mutation spectrums between duodenum and jejunum/ileum cancers.

## Methods

### Clinical specimens

The Formalin-Fixed Paraffin-Embedded (FFPE) tissues samples and blood samples from 298 patients with small bowel cancer (Table 1) collected during 2018 to 2023, were used for this study. These samples had been analyzed using NGS. The analysis had been carried out in a laboratory accredited by the College of American Pathologists (CAP) and Clinical Laboratory Improvement Amendment (CLIA) (3D Medicines Inc., Shanghai, China). DNA-based NGS panel which covers full coding sequence of 733 tumor-related genes, was used for detection of gene mutation in this study. Genomic DNA extraction, targeted panel sequencing were performed as previously reported by our group [9–11].

**Table 1:** Basic characteristics of SBC patients in the cohort

Characteristics	N	%	*p*
**Total**	298	100	
**Gender**			
Male	184	61.74	0.0007
Female	114	38.26	
**Age(Median)**	59.5		
≥ 60	149	50.00	1.0000
< 60	149	50.00	
**Location**			
Duodenum	268	89.93	*p* < 0.0001
Jejunum/Ileum	30	10.07	
***KRAS mutation***			
Without (*KRAS* WT)	146	49.00	0.7773
With (*KRAS* Mu)	152	51.00	
***TP53* mutation**			
Without (*TP53* WT)	140	47.00	0.3961
With (*TP53* Mu)	158	53.00	

### Tissue processing and genomic DNA extraction

Formalin-fixed paraffin-embedded (FFPE) tissue sections were evaluated for tumor cell content using hematoxylin and eosin (H&E) staining. Only samples with a tumor content of ≥ 20% were eligible for subsequent analyses. FFPE tissue sections were placed in a 1.5 microcentrifuge tube and deparaffinized with mineral oil. Samples were incubated with lysis buffer and proteinase K at 56° C overnight until the tissue was completely digested. The lysate was subsequently incubated at 80 °C for 4 h to reverse formaldehyde crosslinks. Genomic DNA was isolated from tissue samples using the ReliaPrep™ FFPE gDNA Miniprep System (Promega) and quantified using the Qubit™ dsDNA HS Assay Kit (Thermo Fisher Scientific) following the manufacturer’s instructions.

### Library preparation and targeted capture

DNA extracts (30–200 ng) were sheared to 250 bp fragments using an S220 focused-ultrasonicator (Covaris). Libraries were prepared using the KAPA Hyper Prep Kit (KAPA Biosystems) following the manufacturer’s protocol. The concentration and size distribution of each library were determined using a Qubit 3.0 fluorometer (Thermo Fisher Scientific) and a LabChip GX Touch HT Analyzer (PerkinElmer) respectively.

For targeted capture, indexed libraries were subjected to probe-based hybridization with a customized NGS panel targeting 733 cancer-related genes, where the probe baits were individually synthesized 5′ biotinylated 120 bp DNA oligonucleotides (IDT). Repetitive elements were filtered out from intronic baits according to the annotation by UCSC Genome RepeatMasker [12]. The xGen® Hybridization and Wash Kit (IDT) was employed for hybridization enrichment. Briefly, 500 ng indexed DNA libraries were pooled to obtain a total amount of 2 μg of DNA. The pooled DNA sample was then mixed with human cot DNA and xGen Universal Blockers-TS Mix and dried down in a SpeedVac system. The Hybridization Master Mix was added to the samples and incubated in a thermal cycler at 95℃ for 10 min, before being mixed and incubated with 4 μl of probes at 65℃ overnight. The target regions were captured following the manufacturer’s instructions. The concentration and fragment size distribution of the final library were determined using a Qubit 3.0 fluorometer (Thermo Fisher Scientific) and a LabChip GX Touch HT Analyzer (PerkinElmer) respectively.

### DNA sequencing, data processing, and variant calling

The captured libraries were loaded onto a NovaSeq 6000 platform (Illumina) for 100 bp paired-end sequencing. Raw data of paired samples (an FFPE sample and its normal tissue control) were mapped to the reference human genome hg19 using the Burrows-Wheeler Aligner (v0.7.12) [13]. PCR duplicate reads were removed and sequence metrics were collected using Picard (v1.130) and SAMtools (v1.1.19), respectively. Variant calling was performed only in the targeted regions. Somatic single nucleotide variants (SNVs) were detected using an in-house developed R package to execute a variant detection model based on binomial test. Local realignment was performed to detect indels. Variants were then filtered by their unique supporting read depth, strand bias, base quality as previously described [14]. All variants were then filtered using an automated false positive filtering pipeline to ensure sensitivity and specificity at an allele frequency (AF) of ≥ 5%. Single-nucleotide polymorphism (SNPs) and indels were annotated by ANNOVAR against the following databases: dbSNP (v138), 1000Genome and ESP6500 (population frequency > 0.015). Only missense, stopgain, frameshift and non-frameshift indel mutations were kept. Copy number variations (CNVs) and gene rearrangements were detected as described previously [14].

### PD-L1 expression by immunohistochemistry (IHC) 22C3 antibody

FFPE tissue sections were subjected to assessment for PD-L1 expression using the PDL1 IHC 22C3 pharmDx assay (Agilent Technologies). PD-L1 expression was determined using Tumor Proportion Score (TPS), the proportion of viable tumor cells showing partial or complete membrane PD-L1 staining at any intensity.

### Tumor Mutational Burden (TMB)

TMB was defined as the number of nonsynonymous and synonymous somatic SNVs and indels in examined coding regions, with driver mutations excluded. All SNVs and indels in the coding region of targeted genes, including missense, silent, stop gain, stop loss, in-frame and frameshift mutations were considered.

### Microsatellite instability (MSI)

One hundred microsatellite loci were selected for MSI determination and for each assay, the top 30 loci with the best coverage were included for the final MSI score calculation. An in-house developed R script was employed to evaluate the distribution of read counts among various repeat length for each microsatellite locus of each sample. The model for determining the stability of each locus is described as follows:

$$P\left(X={n}_{i}\right)={C}_{{N}_{i}}^{{n}_{i}}{{p}_{i}}^{{n}_{i}}{(1-{p}_{i})}^{{N}_{i}-{n}_{i}},$$

where i is the locus being examined, pi stands for the cumulative percentage at the cut-point repeat length (Ci) of the MSS subtype, ni denotes the number of unstable reads, and Ni represents the total number of reads for that locus. A locus was considered unstable if the probability of P (X ≥ ni) was ≤ 0.001. A MSI score was defined as the percentage of unstable loci. Any sample with a MSI score of ≥ 0.4 was classified as MSI-H, and otherwise MSS.

### Statistical analysis

Data analyses were performed using R (version 4.0.5, 2021) and the GraphPad Prism software (version 7.01). Data were presented as the mean ± standard error of the mean (SEM). Differences between the two groups were analyzed using the unpaired Student t Test or an unpaired t Test with Welch’s correction [15, 16]. Analysis of variance was used to investigate more than two groups. Statistical significance was set to a *P* value of less than 0.05. The chi-square or Fisher’s exact tests were used to determine the differences in the PD-L1 expressions, the MSI proportions among patients with small bowel cancel in different primary sites.

## Results

### Genetic profile of Chinese patients with SBC

**Fig. 1:**
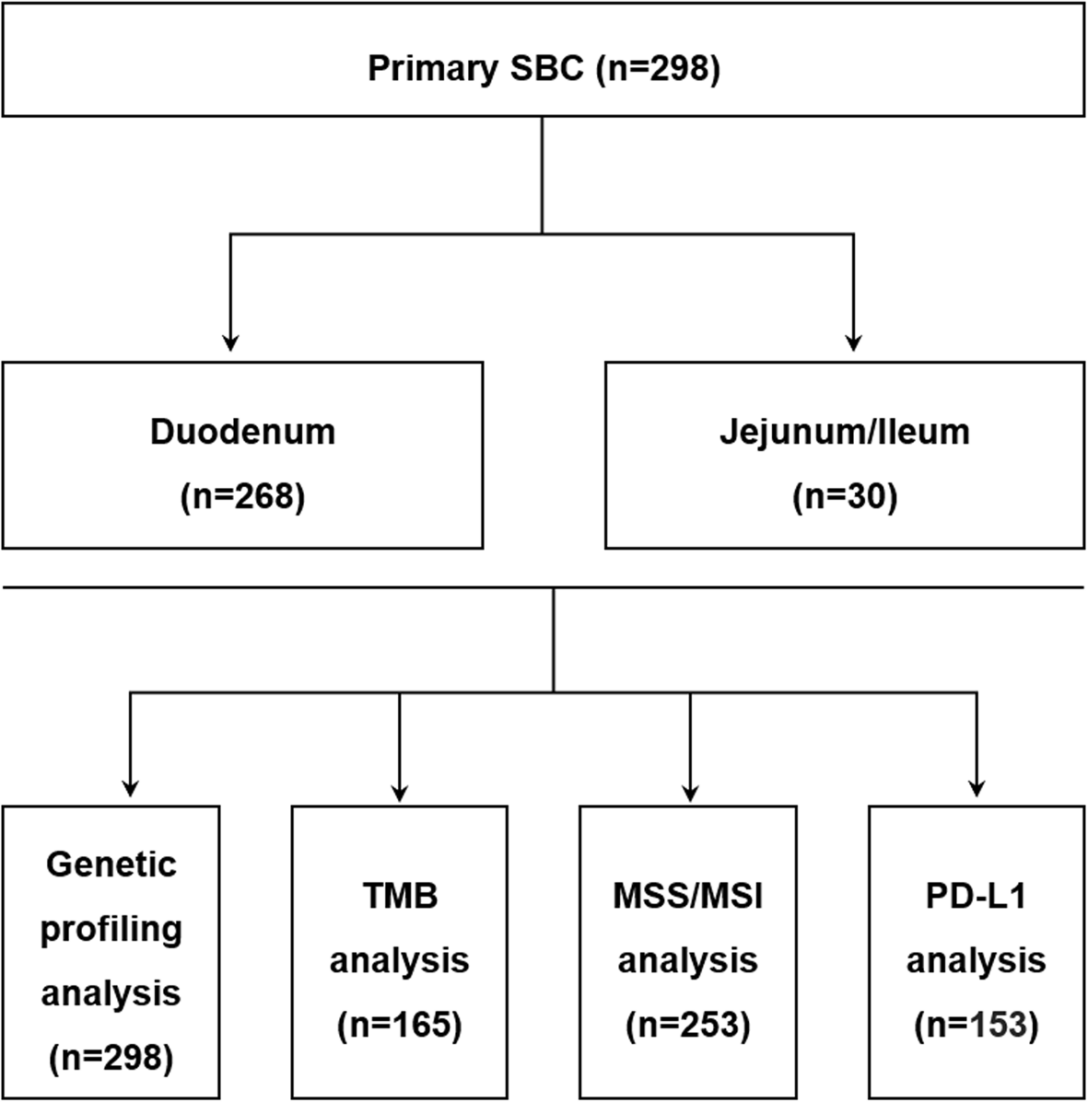
Flow charts for data analysis in this research

**Fig. 2:**
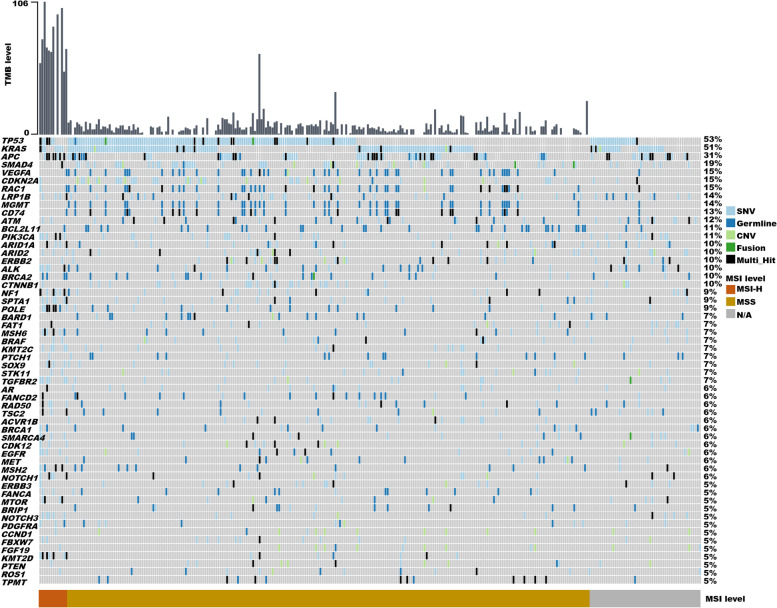
Heatmap showing the landscape of genomic alterations in baseline tissue samples of 298 patients with small bowel cancer

In total, gene testing data of 298 patients were analyzed in this study (Table 1), including 268 patients with primary tumor originating in duodenum, and 30 were in jejunum/ileum (Fig. 1). The genetic profile of 298 patients with SBC was evaluated in this study. The results unveiled that alteration in *TP53* (53%), *KRAS* (51%), *APC* (31%) gene stood out as the most prevalent mutations observed (Fig. 2).

### The frequency of gene mutations in Chinese patients with SBC was observed to be distinct from those present in the MSKCC cohort

**Fig. 3:**
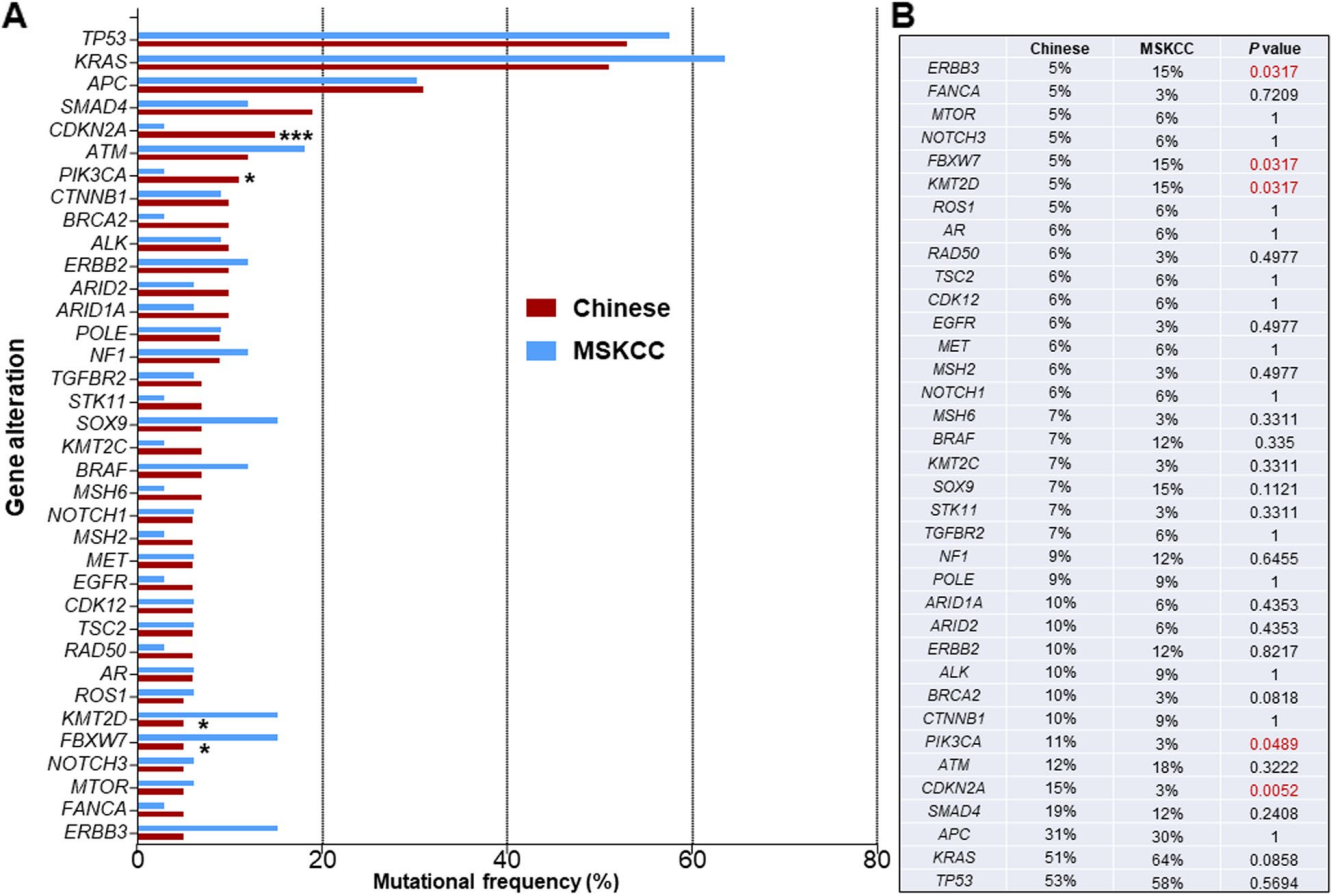
Comparison of mutational frequencies between Chinese and SBA cohorts in MSKCC. **A** Mutation frequency of 36 genes both in Chinese cohort and MSKCC cohort. **B** Statistical analysis of mutational frequency between two cohorts

A comprehensive comparison of mutations in a total of 36 genes was conducted between the Chinese cohort and MSKCC cohort. In contrast to the previously published MSKCC data [17], Chinese patients with SBC exhibited unique mutation characteristics. For instance, the mutation frequencies of genes such as *ERBB2, FBXW7, KMT2D* were comparatively lower, while the mutation frequencies of PIK3CA and CDKN2A were higher (Fig. 3).

### Different gene mutational frequencies were observed among SBC patients, depending on the primary tumor location within the small bowel, such as the duodenum, jejunum, ileum

**Fig. 4:**
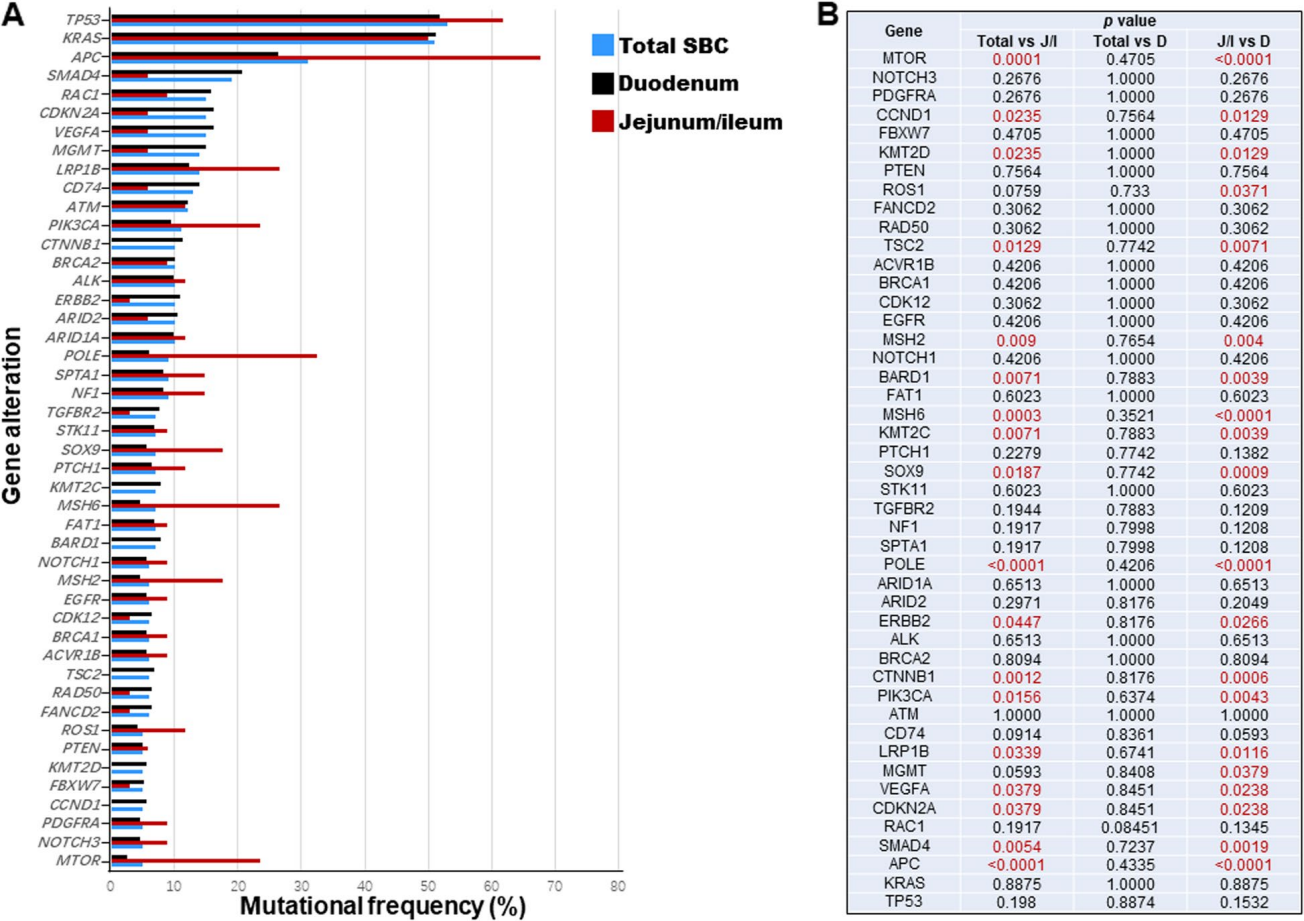
Comparison of mutational frequencies among SBC patients with primary tumor in different sites, including duodenum, jejunum and ileum. **A** comparation of 46 genes mutational frequency among SBC patients with tumor located in duodenum, jejunum, ileum. **B** Statistical analysis of mutational frequency

Small intestine cancer patients with tumors occurring at different anatomical sites also displayed varying molecular mutation characteristics.. Comparison of mutation frequencies across 46 genes among different groups revealed notable differences in tumor mutation genes between the duodenum and the jejunum/ileum.. In particular, the mutation frequencies of genes such as APC, LRP1B, PIK3CA, POLE, MSH6, MTOR, and others were significantly higher in the jejunum or ileum group compared to the duodenum group; while the duodeum group displayed higher mutation frequencies in genes like SMAD4, CDKN2A, VEGFA, CTNNB1, ERBB2, etc. (Fig. 4).

### Differences in the level of biomarkers related to the efficacy of immunotherapy were shown in SBC patients with tumors occurring in different anatomical sites

**Fig. 5:**
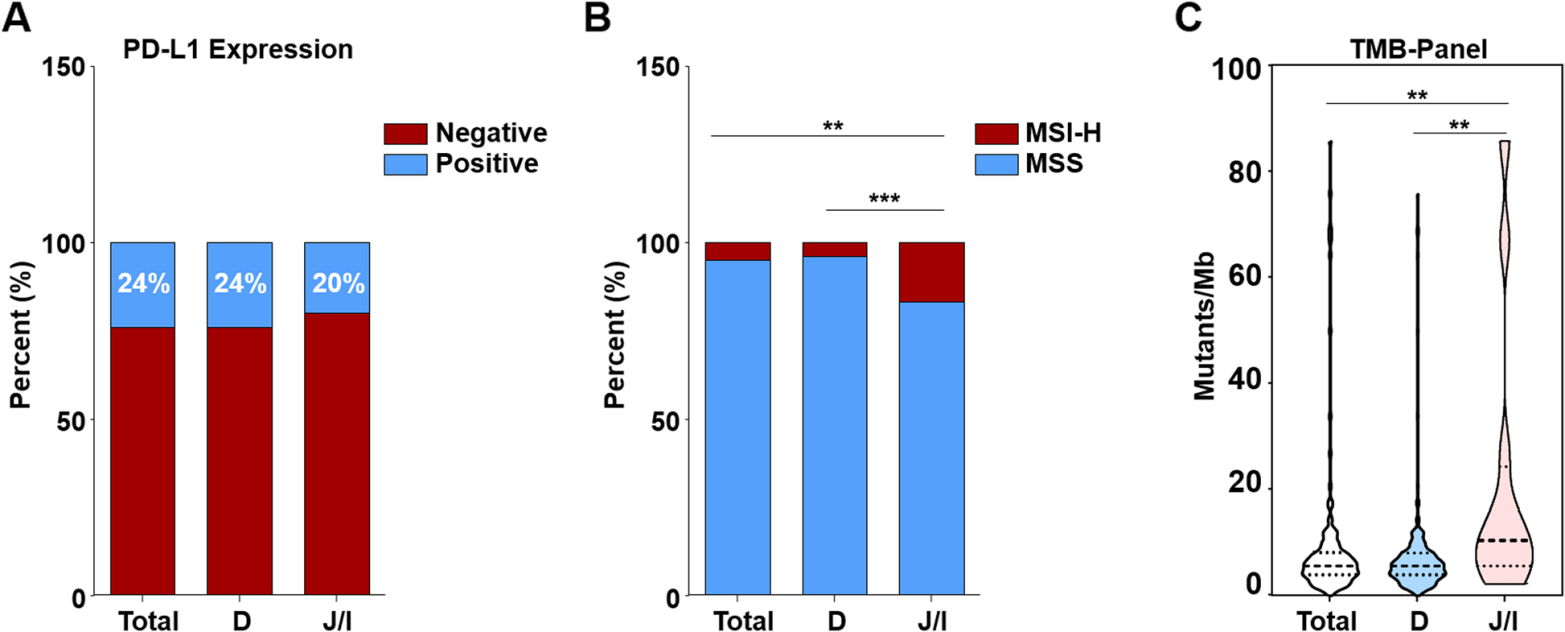
Analysis of biomarkers related to the efficacy of immunotherapy. **A** comparation of PD-L1 expression level among patients with primary tumor located in duodenum, jejunum and ileum. **B** comparation of MSI-H proportion among patients with primary tumor located in duodenum, jejunum and ileum. **C** comparation of TMB level among patients with primary tumor located in duodenum, jejunum and ileum

PD-L1 expression level, proportion of MSI-H, and TMB levels were compared among SBC patients with tumor occurring in different anatomical sites, including duodenum, jejunum, and ileum. The findings indicated that while there was no significant difference in PD-L1 expression level amongst these groups, SBC patients with primary tumors located in the jejunum or ileum displayed higher proportions of MSI-H and TMB levels compared to those with tumors in duodenum (Fig. 5).

## Discussion

The small intestine, comparising the duodenum, jejunum, and ileum, constitutes 75% of the length and 90% of the functional absorptive capacity of gastrointestinal tract, yet only account for 2%-6% of all gastrointestinal tract malignancies, as reported in literature [1, 2, 18]. The rarity of SBC has resulted in limited research on the genetic landscape. Previous researches have mainly focused on the comparison of genetic characteristics between SBC and colorectal cancer (CRC)/gastric cancer(GC) [7], or the results based on smaller sample sizes [8]. In this study, we analyzed the genetic characteristics of SBC in a chort of 298 Chinese patients with SBC. Our analysis compared these results with SBC-specific data, rather than CRC/GC, as reported by MSKCC [17].

The association of tumor location in CRC with molecular and clinical heterogeneity was widely demonstrated, especially the difference in genetic landscape between left and right CRC has been extensively and multi-dimensionally analyzed in the last decade [19–21]. Moreover, recent researchers have found that the immunologic characteristics were also different between left and right CRC [20, 22]. All these differences eventually affected the efficacy of CRC therapy such as ICIs treatment [23, 24]. These findings suggest that heterogeneity in SBC may also be influenced by tumor location. However, due to limited data in previous research, further investigation is warranted to confirm this observation. Consequently, we conducted an analysis of genetic characteristics, including mutant genes, TMB level, MSI/MSS, and PD-L1 expression. Our results revealed dignificant differences in mutation frequencies of certain genes between duodenum and jejunum/ileum cancers, specifically in APC, SMAD4, CDKN2A, PIK3CA, etc. This observation suggests that the varying responses to treatment in relation to mutated genes could potentially account for the heterogeneous clinical outcomes. For example, it has been demonstrated that alteration in SMAD4 was associated with resistance to chemotherapy in CRC [25]. Our findings indicated a higher proportion of SMAD4 mutation in duodenal cancer patients in comparison to those with jejunum or ileum cancers. This may suggest different response or resistance to chemotherapy and this guide future exploration.

The development and clinical applicability of ICIs such as anti-PD-1/PDL-1 signify an important milestone in the therapy of solid tumors. Notably, low response rate to ICI treatments were found in patients who were not pre-screened for certain biomarkers, particularly in the context of gastrointestinal cancers [26]. Nowadays, various ICIs, including pembrolizumab and nivolumab, have received approval for treating patients with mismatch-repair deficiency or microsatellite instability (dMMR/MSI-H), given the reports on significant improvement in response rate and clinical benefits shown in clinical trials [26–30]. Meanwhile, the efficacy of ICIs for dMMR/MSI-H SBC has also been confirmed in KEYNOTE-158, a phase II study of Pembrolizumab in patients with previously treated, advanced non-colorectal MSI-H/dMMR cancer [29, 31, 32]. It has to be noted that based on a better clinical outcome in a specific patient subset with TMB-H solid tumors (≥ 10 mutations/mega-base (mut/Mb)), spanning 9 different tumor types enrolled in KEYNOTE-158, TMB has also been approved by FDA as a predictor of ICIs treatment for pan-solid tumors [33]. In addition, the expression level of PD-L1 was another predictive biomarker of ICIs therapy for several solid tumors, including non-small cell lung cancer [34], gastroesophageal cancer [35], etc. Therefore, here we analyzed microsatellite state, level of TMB, and PD-L1 expression in Chinese patients with SBC, and compared differences in these biomarkers between duodenum cancer and jejunum/ileum cancer. Our findings indicate that, although no significant differences were found in PD-L1 expression among Chinese SBC patients as a whole,, as well as between those with duodenum cancer and jejunum/ileum cancer subtypes, there was a higher proportion of dMMRMSIH and TMB levels in patients with jejunum/ileum cancer as compared to duodenum cancer (Fig. 5). According to the above descriptions, it is plausible to suggest that the SBC patients with different tumor locations may exhibit different responses to ICI therapy.

Moreover, the different genetic characteristics observed in Chinese and MSKCC cohorts, as well as within the jejunum/ileum cancer and duodenum cancer subgroups, may indicate variations in the prognosis of these patients. The investigators have discovered that the suppresion of CDKN2A expression could inhibit tumor cell proliferation and diminish the epithelial-mesenchymal transition (EMT) progression in CRC. Therefore, it is plausible that CDKN2A induced-EMT could be linked to metastasis of CRC [36]. Our results showed a greater frequency of CDKN2A mutation in SBC patients of our cohort compared to that of the MSKCC cohort report (Fig. 3), Conversely, there was a lower proportion of CDKN2A mutation in jejunum/ileum patients as compared to duodenum patients (Fig. 4). The aforementioned findings suggest that there could exist varying prognoses and degrees of metastatic risk in Chinese SBC patients in comparison with the western SBC population. Additionally, patients diagnosed with jejunum/ileum cancer vensus duodenum cancer may similarly exhibit different prognoses and metastatic risk.

Several confounding factors, such as geographical location, environmental factors, and race, may potentially impact the genetic mutation factors. Moreover, various literature reports have shown that the co-mutation of two or more genes may impact treatment decisions and prognosis for patients. For instance, the co-mutation of KRAS/TP53 and EGFR has been shown to have an impact on the effectiveness of EGFR-TKIs [37, 38]. Similarly, the co-mutation of TP53 and KEAP1 may affect the effectiveness of immunotherapy, as reported in lung and colorectal cancers [39, 40]. Hence, we should pay attention to such factors. However, due to the limited sample size in this study, further analysis could not be carried out, and this is an area that we must explore in the future.

## Conclusion

Patients with duodenal cancer and those with cancer occurring in the other region of the small bowel displayed obvious differences in gene landscape. Consequently, it is recommended that these patients undergo distinct treatment strategies. Especially, the level of biomarkers for immunotherapy, such as MSI-H, and PD-L1 expression, were also different among SBC patients with tumors originating from different anatomical sites. The implications of these findings suggest that these patients may exhibit different responses to immunotherapy.

### Supplementary Information

**Additional file 1: Table S1.** List of genes of the 733-gene panel.

## Data Availability

The data and materials used and/or analysed during the current study available from the corresponding author on reasonable request.
