# Supplementary material for: Genetic profile of Chinese patients with small bowel cancer categorized by anatomic location
Source: BMC Med Genomics. 2023 Nov 16;16:289. doi: 10.1186/s12920-023-01736-z (PMC10652443; doi:10.1186/s12920-023-01736-z)
Supplement: Supplementary file 1 — Additional file 1: Table S1. List of genes of the 733-gene panel. [file 12920_2023_1736_MOESM1_ESM.pdf]

Table S1. List of genes of the 733-gene panel

ABL1	CDX2	FGFR4	MLH1	PTEN	VEGFA	JMJD1C	TRIM37	BCL11A	EZR	TBL1XR1	PLXNB1	LIG1	RNF168	POLD3
ACVR2A	CHD2	FH	MLLT3	PTK6	VHL	LMO1	TSHR	BCL11B	FAT4	TCF7L2	SPRED1	LIG3	RNF4	POLD4
AFF3	CHEK1	FHIT	MPL	PTPRD	NSD3	LZTR1	UROD	BCORL1	FUBP1	TCL1A	ERF	LIG4	RNF8	POLE2
AKT1	CHEK2	FLCN	MRE11A	RAC1	ZNF479	MAX	WAS	BIRC3	FUS	TET1	RPS6KA3	MAD2L<br>2	RPA1	POLE4
AKT2	CHIC2	FLT1	MSH2	RAD50	ZNRF3	MEN1	WRN	BRD4	GAS7	TFE3	GSK3B	MBD4	RPA2	PPP4R1
AKT3	CIC	FLT3	MSH3	RAD51	ABCB11	MTAP	WT1	CACNA1<br>D	H3F3A	TNFAIP3	NOTCH3	MDC1	RPA3	PPP4R3A
ALK	CIITA	FLT4	MSH6	RAD51C	APOBEC3<br>B	MUTYH	XPA	CALR	HIF1A	USP8	NOTCH4	MGMT	RPA4	PPP4R3B
ANK1	CRBN	FOXA1	MTOR	RAF1	AXIN2	NBN	XPC	CAMTA1	HIP1	WIF1	ALKBH2	MLH3	RRM2B	PPP4R4
APC	CRLF2	FRS2	MYC	RARA	BARD1	NHP2	XRCC2	CANT1	HNRNPA2B<br>1	XPO1	ALKBH3	MMS19	SETMAR	RAD9B
AR	CRNKL1	G6PD	MYCN	RB1	BMPR1A	NME1	HOXB13	CARD11	HOXA11	ZFHX3	APEX1	MNAT1	SEM1	RBX1
ARAF	CRTC3	GATA3	MYD88	RET	BUB1B	NOP10	BCL2L1	KNL1	IL6ST	ACVR1B	APEX2	MPG	SHPRH	RFC1
AREG	CSF1R	GLI2	NF1	RGS7	CDC73	NTHL1	BCL6	CASP8	KDM6A	ARID1B	CENPS	MSH4	SMUG1	RFC2
ARHGAP<br>5	CSF3R	GNA11	NF2	RICTOR	CDKN1C	PHOX2B	CDK8	CBFA2T3	KEAP1	DNMT1	APLF	MUS81	SPO11	RFC3
ARID1A	CTNNB1	GNAQ	NFE2L2	RNF43	CEBPA	PMS1	FOXP1	CBFB	KLF4	FOXL2	APTX	NEIL1	TDG	RFC4
ARNT	CTNND2	GNAS	NFIB	ROS1	COL7A1	POLH	GRIN2A	CBLB	LCK	GATA1	ATRIP	NEIL2	TDP1	TELO2
ASXL1	CUL3	HDAC2	NKX2-1	RPTOR	CTR9	POLQ	IKBKE	CCDC6	LEF1	HIST1H3<br>B	FAAP100	NEIL3	TDP2	TIMELES<br>S
ATM	CYSLTR<br>2	HEY1	NOTCH1	RUNX1	CXCR4	POT1	MEF2B	CCNB1IP<br>1	LIFR	KDM5C	FAAP24	NHEJ1	TOP3A	TMEM189
ATR	DDR2	HGF	NOTCH2	SDC4	CYLD	PRDM9	NFKBIA	CD79A	MAPK1	MAP3K1	FAAP20	NUDT1	TOP3B	WDR48

AURKA	DICER1	HOOK3	NPM1	SDHC	DDB2	PRF1	PIK3CD	CD79B	MED12	KMT2C	MPLKIP	NABP2	TOPBP1	GFI1
AXL	DNMT3A	HRAS	NRAS	SERPINB3	DIS3L2	PRKAR1A	SRC	CDH11	NAB2	NCOR1	CCNH	OGG1	TP53BP1	CYP17A1
B2M	DPYD	IDH1	NRG1	SETD2	DKC1	PRSS1	BTG1	CHD4	NCOR2	PHF6	CDK7	PARP1	TREX1	ELF3
BAP1	EGFR	IDH2	NTRK1	SF3B1	DOCK8	PTPN11	DIS3	CLIP1	NDRG1	PPP2R1A	CETN2	PARP2	TREX2	SGK1
BAZ1A	EPHA2	IGF1R	NTRK2	SH2B3	DROSHA	PTPN13	EED	CLTCL1	NONO	PRDM1	CHAF1A	PARP3	UBE2A	GSTT1
BCL2	EPHA3	IGF2	NTRK3	SLC29A1	ELANE	RAD51B	GNA13	CNBP	PAX3	SOCS1	CLK2	PCNA	UBE2B	AEN
BCOR	ERBB2	IL7R	PAK1	SMAD4	EPCAM	RAD51D	NT5C2	CNOT3	PAX7	SOX9	DCLRE1A	PNKP	UBE2N	CCNO
BLM	ERBB3	INPP4B	PALB2	SMARCA1	ERCC3	RECQL	PPP2R2A	CREB3L1	PAX8	TRAF7	DCLRE1B	POLB	UBE2T	CENPX
BMP5	ERBB4	ITGAV	PAX5	SMARCA4	ERCC5	RECQL4	NSD2	CREB3L2	PER1	IKZF1	DCLRE1C	POLI	UBE2V2	CUL4A
BRAF	ERCC1	JAK1	PBRM1	SMARCB1	ETV6	RFWD3	EPHA7	CREBBP	PICALM	MYCL	DDB1	POLK	UNG	CUL5
BRCA1	ERCC2	JAK2	PDCD1LG2	SMO	EXT1	RHBDF2	GLI1	CRTC1	PIM1	NCOA3	DMC1	POLL	USP1	DNTT
BRCA2	ERCC4	JAK3	PDGFB	SRGAP3	EXT2	SBDS	MYB	CTCF	POU2AF1	CDK2	DUT	POLM	XAB2	ELOA
BRIP1	ERCC6	JUN	PDGFRA	SRSF2	FAH	SDHA	NRG3	CUX1	POU5F1	LATS1	EME1	POLN	XRCC1	HUS1B
BTK	EREG	KCNJ5	PDGFRB	STAG2	FANCD2	SDHAF2	NUP93	DAXX	PPP6C	LATS2	EME2	PRKDC	XRCC3	PER2
CARS	ESR1	KDR	PDPK1	STK11	FANCE	SDHB	PTK2	DDIT3	PRDM16	YAP1	ENDOV	PRPF19	XRCC4	PER3
CBL	EWSR1	KIT	PIK3CA	SUZ12	FANCF	SDHD	RXRA	DDX10	PREX2	TEAD2	ERCC8	RAD1	XRCC5	MSH5
CCND1	EZH2	KMT2A	PIK3CB	SYK	FANCI	SERPINA1	SMARCA2	DDX3X	PRKACA	MGA	EXO1	RAD18	XRCC6	PARP4
CCND2	FAM135B	KMT2D	PIK3R1	TBX3	FANCL	SETBP1	TYK2	DDX5	PTPRT	HES1	FAN1	RAD23A	ABRAXAS1	POLE3

CCND3	FAM47C	KRAS	PIK3R2	TCF3	FANCM	SH2D1A	ZNF750	DDX6	QKI	KDM5A	FANCB	RAD23B	FRK	PPP4R2
CCNE1	FANCA	LASP1	PLCG2	TERT	FAS	SHOC2	ABI1	DNM2	RAD21	SPEN	GEN1	RAD52	BIRC5	SLX1A
CD274	FANCC	LMNA	PML	TET2	FEN1	SLC25A13	ACKR3	EBF1	RANBP2	THBS2	GTF2H1	RAD54B	EMSY	RAD54L2
CDH1	FANCG	LRP1B	PMS2	TMEM127	GALNT12	SLX4	ACSL3	EIF3E	RAP1GDS1	CUL1	GTF2H3	RAD54L	CRKL	RFC5
CDH10	FAT1	MAP2K1	POLD1	TMPRSS2	GATA2	SOS1	ACVR1	EIF4A2	RBM10	HDAC1	GTF2H4	RAD9A	EPHB1	HMGA2
CDK12	FBXW7	MAP2K2	POLE	TOP2A	GBA	SPOP	AFF4	ELF4	RHOA	MLST8	GTF2H5	RBBP8	GLI3	TSPAN31
CDK4	FES	MAP2K4	POLG	TP53	GJB2	SPRTN	AMER1	ELK4	RHOH	PIK3R3	H2AFX	RDM1	IRS2	MYOD1
CDK6	FGF19	MCL1	PPARG	TPMT	GPC3	SRY	ARID2	ELL	RNF213	RHEB	HELQ	RECQL5	RUNX1T1	CHD1
CDKN1A	FGF3	MDM2	PPM1D	TSC1	GREM1	STAT3	ATP1A1	EP300	SFPQ	RPS6KB1	HFM1	REV1	SLIT2	ZBTB16
CDKN1B	FGF4	MDM4	PRCC	TSC2	HFE	SUFU	ATP2B3	EPAS1	SLC34A2	GRB2	HLTF	REV3L	SOX2	PCDH9
CDKN2A	FGFR1	MECOM	PRKCH	U2AF1	HMBS	TGFBR1	ATRX	EPS15	SLC45A3	RIT1	HMGB1	RIF1	SPTA1	PLXNA1
CDKN2B	FGFR2	MET	PSIP1	UGT1A1	HNF1A	TGFBR2	AXIN1	ERC1	SMAD2	RASA1	HUS1	RMI1	ZNF217	
CDKN2C	FGFR3	MITF	PTCH1	USP6	ITK	TP63	BCL10	ETNK1	SMAD3	ERRFI1	UVSSA	RMI2	ZNF703	
